# Circulating metabolic markers after surgery identify patients at risk for severe postoperative complications: a prospective cohort study in colorectal cancer

**DOI:** 10.1097/JS9.0000000000000965

**Published:** 2023-12-18

**Authors:** Blanca Montcusí, Francisco Madrid-Gambin, Óscar J Pozo, Santiago Marco, Silvia Marin, Xavier Mayol, Marta Pascual, Sandra Alonso, Silvia Salvans, Marta Jiménez-Toscano, Marta Cascante, Miguel Pera

**Affiliations:** a Department of Surgery, Section of Colon and Rectal Surgery, Hospital del Mar; b Colorectal Neoplasms Clinical and Translational Research Group; c Applied Metabolomics Research Group, Hospital del Mar Medical Research Institute (IMIM); d Department of Surgery, Faculty of Medicine, Universitat de Barcelona (UB); e Signal and Information Processing for Sensing Systems, Institute for Bioengineering of Catalonia (IBEC), The Barcelona Institute of Science and Technology; f Department of Electronics and Biomedical Engineering, Faculty of Physics; g Department of Biochemistry and Molecular Biomedicine, Faculty of Biology; h Institute of Biomedicine, Universitat de Barcelona (UB); i Department of General and Digestive Surgery, Institut of Digestive and Metabolic Diseases, Hospital Clínic, Barcelona; j CIBER of Hepatic and Digestive Diseases (CIBEREHD), Institute of Health Carlos III (ISCIII), Madrid, Spain

**Keywords:** colorectal cancer, metabolomics, postoperative complications

## Abstract

**Background:**

Early detection of postoperative complications after colorectal cancer (CRC) surgery is associated with improved outcomes. The aim was to investigate early metabolomics signatures capable to detect patients at risk for severe postoperative complications after CRC surgery.

**Materials and methods:**

Prospective cohort study of patients undergoing CRC surgery from 2015 to 2018. Plasma samples were collected before and after surgery, and analyzed by mass spectrometry obtaining 188 metabolites and 21 ratios. Postoperative complications were registered with Clavien–Dindo Classification and Comprehensive Complication Index.

**Results:**

One hundred forty-six patients were included. Surgery substantially modified metabolome and metabolic changes after surgery were quantitatively associated with the severity of postoperative complications. The strongest positive relationship with both Clavien–Dindo and Comprehensive Complication Index (β=4.09 and 63.05, *P*<0.001) corresponded to kynurenine/tryptophan, against an inverse relationship with lysophosphatidylcholines (LPCs) and phosphatidylcholines (PCs). Patients with LPC18:2/PCa36:2 below the cut-off 0.084 µM/µM resulted in a sevenfold higher risk of major complications (OR=7.38, 95% CI: 2.82–21.25, *P*<0.001), while kynurenine/tryptophan above 0.067 µM/µM a ninefold (OR=9.35, 95% CI: 3.03–32.66, *P*<0.001). Hexadecanoylcarnitine below 0.093 µM displayed a 12-fold higher risk of anastomotic leakage-related complications (OR=11.99, 95% CI: 2.62–80.79, *P*=0.004).

**Conclusion:**

Surgery-induced phospholipids and amino acid dysregulation is associated with the severity of postoperative complications after CRC surgery, including anastomotic leakage-related outcomes. The authors provide quantitative insight on metabolic markers, measuring vulnerability to postoperative morbidity that might help guide early decision-making and improve surgical outcomes.

## Introduction

Highlights

- Preoperative metabolome is not associated with the severity of postoperative complications after colorectal cancer surgery.
- Surgery substantially alters the metabolome.
- Circulating metabolic markers reveal severe postoperative complications risk.

Surgery is at present the only treatment with curative intent for patients with colorectal cancer (CRC), whereas chemotherapy is predominantly used in an adjuvant setting^1,2^. Despite improvements in technique and perioperative care, more than 25% of patients develop postoperative complications^3^, which are associated with morbidity and mortality in both the short-term and long-term.

Severe complications consist of anastomotic leakage (AL), ileus, bleeding, and also nonsurgical morbidity^4^. AL is one of the most significant, with reported incidences ranging from 2 to 30% depending on tumor location and leak definition^5^. This complication compromises not only short-term but also long-term outcomes. Several studies have suggested that the consequent peritoneal infection is associated with higher recurrence rates and cancer-specific mortality^6^.

Identifying and early diagnosing patients at risk for severe complications are essential for improving short-term outcomes and facilitating clinical decision-making^7^. C-reactive protein, which is one of the most common used markers for postoperative complications, offers poor specificity, and other proposed markers have not improved its informative value^8^. Hence, there is a need for additional biomarkers capable of specifically detecting patients at risk for severe postoperative complications, in order to establish prevention and/or early detection strategies.

Quantitative metabolomics includes describing the metabolic composition of a sample in terms of metabolite presence and concentration^9^. Metabolites change dynamically in response to disease and treatment. Metabolic phenotyping is one of the most widely applicable fields for the evolution of precision medicine, concerning clinical diagnosis, treatment selection, and prognosis, and is being implemented in hospital environments^10,11^. Patients undergoing surgery are exposed to major trauma, environmental drugs, bacteria, and nutritional strategies; therefore, it is conceivable that surgery may alter the metabolome^12^. Characterization of the metabolic fingerprint during the perioperative period has been used to predict surgical outcomes after cardiac surgery, liver and kidney transplant, and bariatric surgery^13–16^.

Regarding CRC surgery, some studies have focused on determining markers for postoperative complications. Ischemia-modified albumin turned out to be a biomarker for AL and citrulline for prolonged ileus^17^. Elevated intestinal fatty acid binding protein could also be used for AL risk assessment, preoperatively in serum^18^ and postoperatively in urine^19^. Urinary and peritoneal fluid compounds, such as volatile organic compounds^20^ and neopterin^21^, were considered potential biomarkers for AL. A few studies exhibited reduced lysophosphatidylcholine (LPC), phosphatidylcholine (PC), and asymmetric dimethylarginine levels in the blood of patients with complications after CRC surgery^22,23^. Overall, these studies reinforce the theory that the application of metabolomics helps in identifying new biomarkers that could anticipate CRC postoperative complications. Extending this knowledge to specific species and the saturation degree of fatty acids of LPCs and PCs, along with amino acids other than citrulline and arginine, appears promising in establishing the risk and the severity of such complications.

Our aim was to assess the ability of broad-based, targeted metabolomics to identify patients at risk for severe postoperative complications following CRC surgery. We evaluated the impact of surgery on the metabolome, and the association of pre and postoperative metabolic markers with the severity of complications, with an emphasis on AL. We hypothesized that metabolomics would reliably prognosticate the risk of severe complications, which would be of use to clinical practice.

## Materials and methods

### Study design and participants

This single-center observational study included a prospective cohort of patients who underwent CRC surgery between October 2015 and May 2018. The study was approved by the Ethical Committee and it was registered at ClinicalTrials.gov. All patients provided written informed consent. The work was reported in line with the strengthening the reporting of cohort studies in surgery (STROCSS) criteria^24^ (Supplemental Digital Content 1, http://links.lww.com/JS9/B612). Patients with stage IV cancer, receiving neoadjuvant therapy, noncurative resection or urgent surgery were excluded.

We collected BMI, smoking, alcoholism, arterial hypertension, diabetes mellitus, dyslipidemia, and anticoagulant therapy. All patients received antibiotic prophylaxis, and those undergoing proctectomy also received mechanical bowel preparation. The same surgical team performed all operations and perioperative outcomes were registered, along with tumor location, surgical approach, type and duration of operation, perioperative transfusion, and length of stay. Postoperative complications were recorded within 30 days after surgery and classified in accordance with the Clavien–Dindo Classification (CDC)^25^, a system that has seen extensive use and has been validated in hundreds of studies across many fields of surgery. Furthermore, the Comprehensive Complication Index (CCI)^26^ was also calculated in order to better report and summarize the overall morbidity burden on a numerical scale (0–100). Patients were divided into those presenting ‘major’ postoperative complications (defined as CDC ≥ IIIa or CCI ≥26.2) or ‘minor’ (defined as CDC <IIIa or CCI <26.2), based on previous works^26,27^. Patients were considered to have an intra-abdominal infection (intra-abdominal abscess or AL with or without peritonitis) when clinical suspicion was confirmed with radiologic or surgical exploration. The intra-abdominal abscess was defined as a postoperative intra-abdominal fluid collection usually treated with antibiotics or drainage.

### Metabolomics analysis

Six-hour fasted venous blood samples were collected before surgery and on postoperative day 4. Plasma metabolic profiles were determined using the AbsoluteIDQ p180 kit (Biocrates Life Sciences AG)^28^. This kit quantifies 188 endogenous metabolites from six different compound classes, including most of those previously reported as associated with postoperative complications; that is, PCs, LPCs, and amino acids. The sample plate was processed according to the manufacturer’s instructions. Analyses were performed in the AB Sciex 4000 QTRAP MS/MS mass spectrometer (AB Sciex LLC) coupled to an Agilent 1260 Infinity HPLC system (Agilent). Analyst (Sciex) and the MetIDQ software packages were used to analyze the obtained data and calculate metabolite concentrations, as stated in µM. Several ratios between metabolites were performed to obtain valuable information about targeted metabolic processes (Supplementary Table 1, Supplemental Digital Content 2, http://links.lww.com/JS9/B613).

### Statistical analysis

This is an exploratory observational study in which we did not carry out a formal calculation of the sample size. The absence of published results on the changes in the metabolic phenotype in plasma after colorectal surgery prevented the calculation. Instead, we included all patients who met the inclusion criteria during the study period.

Data preprocessing and analysis were conducted using the R software version 4.2. Metabolic markers with more than 80% of samples below the limit of quantification were removed from the final dataset. Missing values were imputed using the Probabilistic Principal Component Analysis. The dataset was log-transformed before modeling.

Potential associations of preoperative metabolic markers with CDC and CCI were assessed using multiple linear regression (MLR). Each metabolic marker was assessed individually. Models were adjusted for age, sex, BMI, smoking, alcoholism, arterial hypertension, diabetes mellitus, dyslipidemia, and anticoagulant therapy. To control for multiple comparison, the Benjamini–Hochberg procedure was carried out on all analyses and the false discovery rate (FDR) was calculated (Benjamini and Hochberg, 1995). An FDR-corrected *P*-value <0.05 was considered statistically significant. Likewise, multivariate modeling for both CDC and CCI was performed, separately, on Pareto-scaled data using a double cross-validated partial least squares (PLS) regression^29^. Covariates were included in the predictor block.

The impact of surgery on the metabolome was tested using a generalized linear mixed model (GLMM) adjusted for covariates, in order to control the individual variability as a random effect. Associations of the metabolic dysregulation with CDC and CCI were carried out by deducting preoperative from postoperative data (Δ-data). Additional covariates from surgery were added to MLR models. Metabolic changes were additionally explored using a multilevel-PLS, as a complementary multivariate approach. More details about univariate and multivariate modeling are specified in the supplementary material (Supplemental Digital Content 2, http://links.lww.com/JS9/B613).

Odds ratios (OR) were calculated from the generalized linear models (GLM) with binomial distribution using both CDC and CCI stratifications, and metabolic variables to assess the risk of severe postoperative complications using only postoperative samples. Polar plots were acquired illustrating ORs of significant variables. The receiver operating characteristic (ROC) analyses on the most relevant compounds were performed, and the optimal concentrations that maximize the sensitivity and specificity of ROC curves were calculated. Optimal concentration cut-offs were used to establish a risk threshold. After stratifying markers by their cut-off values, associations with the complication indexes were newly assessed and adjusted for covariates. The area under the curves (AUCs) and corresponding 95% CI, ORs with 95% CI and *P*-values were extracted to determine the potential translational use of these concentration values. Considering AL as one of the most significant complications after CRC surgery, the variable ‘AL-related complications’ was created and considered true when AL, intra-abdominal abscess, peritonitis or septic shock were CDC ≥IIIa. Associations between the stratification by cut-off values and this variable were also calculated.

## Results

### Study participants

During the study interval, 150 patients (91 males and 59 females) were diagnosed with nonmetastatic CRC and considered eligible for the study. Four patients were excluded due to loss to follow-up. Patient characteristics, surgical procedures, and postoperative outcomes of patients are shown in Table 1.

**Table 1:** Baseline characteristics.

	*N*=146
Age, years	71.2±12.2
Sex, Male:Female	89 (61):57 (39)
BMI, kg/m^2^	27.4±4.3
Smoking	
Never	77 (53)
Former	50 (34)
Current	19 (13)
Alcoholism	
No	134 (91)
Yes	12 (9)
Arterial hypertension	
No	52 (36)
Yes	94 (64)
Diabetes mellitus	
No	105 (72)
Type 1	2 (1)
Type 2	39 (27)
Dyslipidemia	
No	80 (55)
Yes	66 (45)
Anticoagulant therapy	
No	133 (91)
Yes	13 (9)
Surgical approach	
Laparoscopic	121 (83)
Open	25 (17)
Type of operation	
Colectomy	119 (81)
Proctectomy	27 (19)
Cancer stage	
Stage I	40 (27)
Stage II	61 (42)
Stage III	45 (31)
CDC	
0	35 (24)
I	42 (29)
II	48 (33)
IIIa	8 (5)
IIIb	8 (5)
IVa	1 (1)
IVb	2 (1)
V	2 (1)
CCI	
<26.2	111 (76)
≥26.2	35 (24)
AL-related complications (CDC ≥IIIa)	
AL	6 (4)
Intra-abdominal abscess	4 (3)
Peritonitis	4 (3)
Septic shock	1 (1)

Values are either mean±SD or *N* (%).

AL, anastomotic leakage; CDC, Clavien–Dindo Classification; CCI, Comprehensive Complication Index.

### Association between preoperative metabolome and the severity of postoperative complications

Results from MLR models denoted no association between any metabolite before surgery and CDC or CCI. All metabolites obtained a FDR-corrected *P*>0.05. Similarly, the PLS models on baseline samples exhibited R^2^ and Q^2^ values of 0.263 and −0.061 for CDC, and 0.366 and −0.018 for CCI, respectively (Supplementary Figs 1A and B, Supplemental Digital Content 2, http://links.lww.com/JS9/B613). The permutation test indicated an inability to predict CDC or CCI (*P*>0.05).

### Effects of surgery on the metabolome

**Figure 1:**
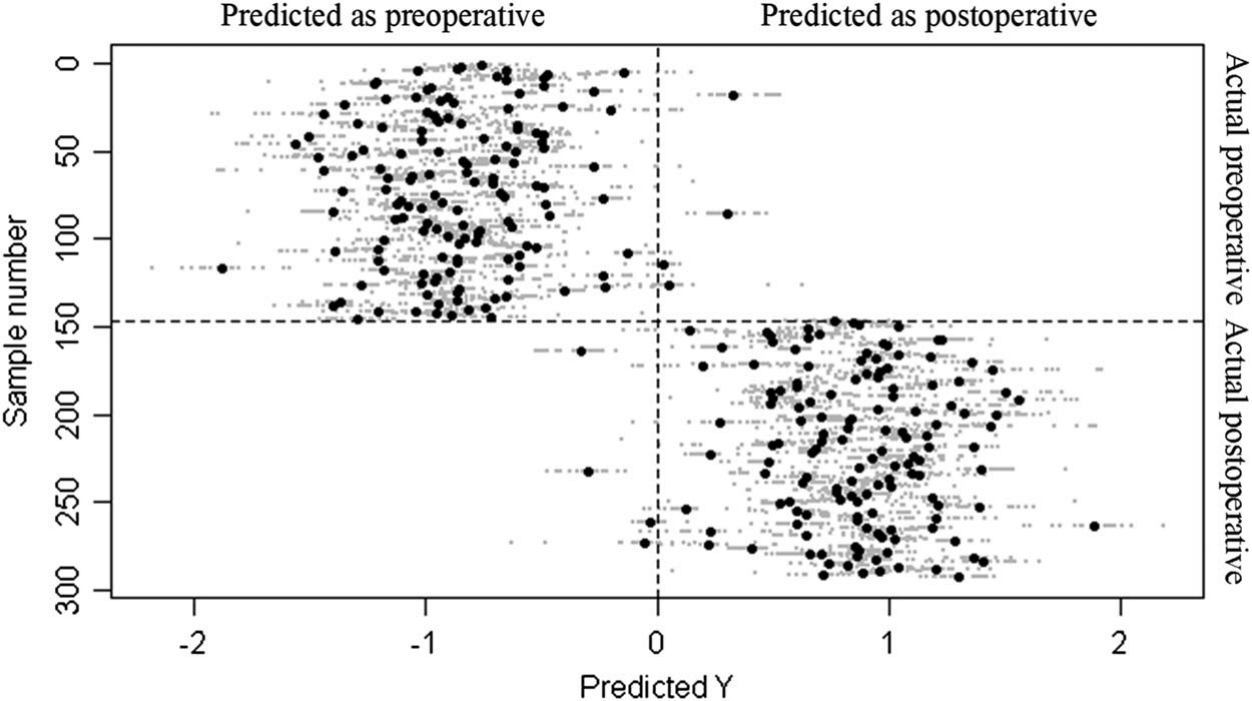
Misclassification plot from multilevel partial least squares discriminant analysis model between preoperative and postoperative samples.

GLMM models exhibited 119 metabolic markers altered after surgery. The multilevel-PLS analysis resulted in a model with a classification rate of 97.3% with a 95% CI of 93.1–99.2 (Fig. 1), with a permutation test *P*<0.001. The results of univariate and multivariate analyses were calculated (Supplementary Table 2, Supplemental Digital Content 2, http://links.lww.com/JS9/B613).

Differential markers mainly included decreased levels of long-chain unsaturated diacyl-PCs (PCa), acyl-alkyl-PCs (PCe), and LPCs; except for PCa34:1, PCa34:2, and PCa32:0 that raised after surgery. Likewise, long-chain sphingomyelin (SM) levels (species 22 to 26) decreased after surgery; while short-chain SM species SM18:0, SM18:1, and SM20:2 increased. There was a prominent rise of the circulating concentration of branched-chain amino acids (BCAAs) and other amino acids, such as methionine, methionine sulfoxide, threonine, phenylalanine, and arginine, among others. The concentration of a few acylcarnitine species also increased following surgery.

### Association between metabolic changes after surgery and the severity of postoperative complications

After adjusting for potential confounders, analysis of Δ-data exhibited 22 and 31 metabolic variables associated with CDC and CCI, respectively. Most of these markers replicated in the PLS analysis are shown in Table 2. The PLS analysis also revealed a strong association of the complication indexes with the type of operation. An additional analysis ruled out the potential association of measured metabolic markers with the surgical approach (all variables, FDR-corrected *P*>0.05). The PLS models exhibited R^2^ and Q^2^ values of 0.435 and 0.155 for CDC, and 0.511 and 0.233 for CCI, respectively; permutation tests validated the model performances (*P*<0.001) (Supplementary Figs 2A and B, Supplemental Digital Content 2, http://links.lww.com/JS9/B613). The strongest positive relationship with both CDC (*β*=4.09, FDR-corrected *P*<0.001) and CCI (*β*=63.05, FDR-corrected *P*<0.001) corresponded to the kynurenine/tryptophan ratio (Supplementary Figs 3A and B, Supplemental Digital Content 2, http://links.lww.com/JS9/B613). An inverse relationship between LPCs and diacyl-PCs ratios followed by several LPC and PC species was found. The LPC16:1/LPC16:0 ratio presented a marked direct association with both indexes (*β*=6.53, FDR-corrected *P*=0.016 for CDC; *β*=106.65, FDR-corrected *P*=0.002 for CCI). Several SM species and circulating threonine concentration also presented an inverse association with both CDC and CCI, while putrescine and methionine sulfoxide had a positive relationship.

**Table 2:** Metabolic markers association with Clavien–Dindo classification and comprehensive complication index on Δ-data.

	CDC				CCI			
Metabolic marker	*P* ^a^	FDR	Beta	LR^b^	*P*	FDR	Beta	LR
Kynurenine/tryptophan	<0.001	<0.001	4.09	2	<0.001	<0.001	63.05	2
LPC16:0	<0.001	0.001	−0.95	1	<0.001	<0.001	−15.34	1
LPC16:0/PCa32:0	<0.001	0.002	−1.06	4	<0.001	<0.001	−17.69	5
LPC18:1/PCe36:1	<0.001	0.004	−1.10	12	<0.001	<0.001	−19.12	10
LPC18:0/PCe36:0	<0.001	0.004	−0.94	13	<0.001	<0.001	−16.38	9
LPC18:1	<0.001	0.004	−0.90	9	<0.001	<0.001	−15.08	4
LPC18:0	<0.001	0.004	−0.91	3	<0.001	<0.001	−14.93	3
LPC18:2	<0.001	0.008	−0.80	11	<0.001	<0.001	−13.30	11
LPC18:2/PCe36:2	<0.001	0.008	−1.02	20	<0.001	<0.001	−17.63	18
SM24:0	0.001	0.016	−1.14	15	0.001	0.012	−15.54	–
LPC16:1/LPC16:0	0.001	0.016	6.53	7	<0.001	0.002	106.65	8
SM(OH)C22:2	0.001	0.018	−1.38	17	0.001	0.008	−20.16	19
Threonine	0.001	0.019	−0.87	8	<0.001	0.002	−14.70	7
Methionine SO	0.002	0.022	1.07	6	0.004	0.030	13.94	14
Kynurenine	0.002	0.022	1.01	10	0.001	0.011	15.02	12
SM(OH)22:1	0.002	0.024	−1.10	21	0.003	0.020	−15.23	–
Putrescine	0.002	0.024	1.96	18	<0.001	0.004	32.40	15
PCe40:1	0.003	0.024	−1.50	–	0.007	0.039	−18.95	–
LPC20:4	0.004	0.036	−0.88	5	<0.001	0.004	−15.30	6
LPC18:2/PCa36:2	0.006	0.055	−2.23	–	0.001	0.008	−38.62	–
Octadecadienylcarnitine	0.007	0.060	−2.07	–	0.004	0.030	-30.89	–
SM24:1	0.009	0.068	−1.09	–	0.005	0.030	−16.50	–
Octadecenoylcarnitine	0.010	0.072	−1.32	–	0.007	0.041	−19.24	–
PCa40:4	0.010	0.072	−1.15	–	0.006	0.038	−17.11	–
LPC18:1/PCa36:1	0.011	0.078	−1.21	14	0.001	0.006	−23.09	13
SM18:1	0.014	0.084	−0.99	19	0.003	0.020	−16.91	16
SM18:0	0.016	0.090	−0.83	–	0.005	0.031	−13.42	20
LPC16:1/PCe32:2	0.016	0.090	−0.73	–	0.001	0.012	−13.43	–
PCe36:4/PCe36:0	0.021	0.107	−0.80	–	0.005	0.031	−13.68	–
Serine	0.026	0.113	−0.62	–	0.001	0.012	−12.48	–
LPC18:0/PCa36:0	0.039	0.137	−0.51	–	0.004	0.030	−9.98	–
LPC17:0	0.098	0.218	−0.67	16	0.038	0.121	−11.74	–
LPC16:1/PCe32:2	0.016	0.090	−0.73	–	0.001	0.01	−13.43	–
PCe385	0.016	0.090	−0.90	–	0.012	0.06	−13.20	–
Isoleucine	0.018	0.095	−0.66	–	0.023	0.09	−8.84	–
PCe36:4/PCe36:0	0.021	0.107	−0.80	–	0.005	0.03	−13.68	–
Hexadecanoylcarnitine	0.022	0.107	−1.53	–	0.016	0.07	−22.40	–

a *P*-values from multiple linear regression models adjusted for age, sex, BMI, smoking, alcoholism, arterial hypertension, diabetes mellitus, dyslipidemia, anticoagulant therapy, surgical approach (laparoscopic versus open) and type (colectomy versus proctectomy) and duration of operation.

b Loading Rank of double cross-validation partial least squares regression. Only significant auto-selected variables are numbered.

CDC, Clavien–Dindo Classification; CCI, Comprehensive Complication Index; FDR, false discovery rate; LPC, lysophosphatidylcholine; PC, phosphatidylcholine; PCa, diacyl-PC; PCe, acyl-alkyl-PC; SO, sulfoxide; SM, sphingomyelin.

### Metabolic markers as potential risk factors for postoperative complications

**Figure 2:**
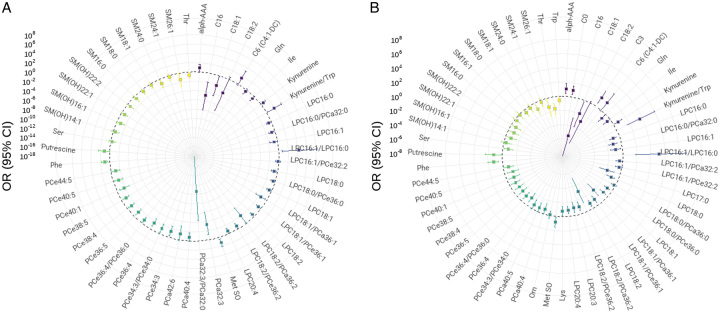
Polar plot illustrating odd ratios and 95% CI of significant markers for dichotomized postoperative complications according to Clavien–Dindo Classification (A) and Comprehensive Complication Index (B).

Stratification based on CDC ≥IIIa resulted in 21 patients with major complications, whereas stratification with CCI ≥26.2 revealed 35 patients with major complications. GLMs were only performed with the results of postoperative samples (rather than Δ-data) in order to facilitate the application in clinical practice. Significant variables from the models, including ORs and 95% CI, are exhibited in the ORs polar plot for dichotomized postoperative complications (Figs. 2A and B). Several metabolic markers reappeared as relevant anew, using only postoperative samples rather than Δ-data. Higher levels of kynurenine/tryptophan and LPC16:1/LPC16:0 ratios were commonly associated with higher ORs for both indexes, along with other metabolites such as alpha-aminoadipic acid and methionine sulfoxide. In contrast, decreases of several LPC, PC, SM species, and certain acylcarnitines, particularly hexadecanoylcarnitine and octadecenoylcarnitine, were associated with higher ORs.

Preoperative and postoperative samples were utilized for an exploratory study of metabolites. However, it is not currently suitable for use in daily clinical practice. In order to overcome this limitation, we exclusively used postoperative samples to investigate the optimal concentration of the metabolic markers that were most strongly associated with severe complications, including LPC16:1/LPC16:0, kynurenine/tryptophan, hexadecanoylcarnitine, and LPC18:2/PCa36:2. We established the concentration of these markers that optimally differed among the risk of presenting major postoperative complications. The optimal cut-off concentrations along with their corresponding AUC (95% CI), OR (95% CI), and *P*-value for all indexes, are shown in Table 3. The ROC curves are shown in Supplementary Figure 4 (Supplemental Digital Content 2, http://links.lww.com/JS9/B613). Patients with circulating levels of LPC18:2/PCa36:2 ratio below the cut-off of 0.081 (µM/µM) resulted in a 4.5-fold higher rate of major complications based on CDC ≥IIIa (0.80 (0.69–0.90) adjusted OR=4.45, 95% CI: 1.52–14.37, *P*=0.008). The same marker stratified by 0.084 value entailed a 7.4-fold higher risk of obtaining a CCI ≥26.2 (adjusted OR=7.38, 95% CI: 2.82–21.25, *P*<0.001). The significant result for the feature AL-related complications implies that LPC18:2/PCa36:2 is associated with the severity of the specific complications included (adjusted OR=5.33, 95% CI: 1.36–26.71, *P*=0.024). The hexadecanoylcarnitine displayed an acceptable statistical performance with a positive association with CDC (adjusted OR=3.71, 95% CI: 1.25–11.88, *P*=0.021), CCI (adjusted OR=5.97, 95% CI: 2.26–17.13, *P*<0.001), and AL-related complications (adjusted OR=11.99, 95% CI: 2.62–80.79, *P*=0.004).

**Table 3:** Likelihood of having Clavien–Dindo classification- and comprehensive complication index-based major complications after surgery relative to remarked metabolic markers concentration.

Outcome	Metabolic marker	Cut-off	Unit	AUC (95% CI)^a^	OR (95% CI)	*P*
CDC	LPC16:1/LPC16:0	≥ 0.026	µM/µM	0.77 (0.66–0.88)	2.55 (0.88–7.84)	0.090
	Kynurenine/tryptophan	≥ 0.062	µM/µM	0.76 (0.64–0.88)	2.82 (0.84–10.14)	0.099
	Hexadecanoylcarnitine	≤ 0.092	µM	0.77 (0.65–0.89	3.71 (1.25–11.88)	0.021
	LPC18:2/PCa36:2	≤ 0.081	µM/µM	0.80 (0.69–0.90)	4.45 (1.52–14.37)	0.008
CCI	LPC16:1/LPC16:0	≥ 0.026	µM/µM	0.81 (0.72–0.90)	5.58 (2.14–15.88)	0.001
	Kynurenine/tryptophan	≥ 0.067	µM/µM	0.83 (0.75–0.91)	9.35 (3.03–32.66)	<0.001
	Hexadecanoylcarnitine	≤ 0.093	µM	0.81 (0.72–0.91)	5.97 (2.26–17.13)	<0.001
	LPC18:2/PCa36:2	≤ 0.084	µM/µM	0.83 (0.75–0.92)	7.38 (2.82–21.25)	<0.001
AL-related complications	LPC16:1/LPC16:0	≥ 0.026	µM/µM	0.83 (0.71–0.95)	3.51 (0.96–15.12)	0.069
	Kynurenine/tryptophan	≥ 0.067	µM/µM	0.83 (0.69–0.94)	4.54 (1.00–23.97)	0.057
	Hexadecanoylcarnitine	≤ 0.093	µM	0.86 (0.76–0.96)	11.99 (2.62–80.79)	0.004
	LPC18:2/PCa36:2	≤ 0.084	µM/µM	0.84 (0.73–0.94)	5.33 (1.36–26.71)	0.024

a AUC and ORs (95% CI), and *P*-values from models adjusted for age, sex, BMI, smoking, alcoholism, arterial hypertension, diabetes mellitus, dyslipidemia, anticoagulant therapy, surgical approach (laparoscopic versus open), type (colectomy versus proctectomy), and duration of operation.

AL, anastomotic leakage; CDC, Clavien–Dindo Classification; CCI, Comprehensive Complication Index; LPC, lysophosphatidylcholine; PC, phosphatidylcholine; PCa, diacyl-PC.

Circulating LPC16:1/LPC16:0 levels higher than 0.026 (µM/µM) significantly increased the risk of CCI-based severe complications (adjusted OR=5.58, 95% CI: 2.14–15.88, *P*=0.001), while it not reached significance neither with CDC (*P*=0.090) nor AL-related complications (*P*=0.069). Similar behavior was found for the kynurenine/tryptophan ratio. Patients with levels above 0.067 (µM/µM) obtained a ninefold higher risk for CCI-based complications (adjusted OR=9.35, 95% CI: 3.03–32.66, *P*<0.001), but no significant findings were reached with CDC (*P*=0.099) or AL-related complications (*P*=0.057).

## Discussion

The present study provides new insights into the relationship between circulating metabolites and vulnerability to severe postoperative complications following CRC surgery. We provide evidence that surgically induced metabolic changes are associated with the severity of short-term postoperative complications. These findings have the potential to contribute in the identification of patients at high vulnerability of severe complications and early decision-making to reduce failure to rescue. Our results also suggest that the preoperative metabolome is not associated with severe postoperative complications. These results are in line with other works^21^, and indicate that metabolites associated with postoperative outcomes are mainly influenced by surgical performance.

We found changes in 119 metabolic markers after CRC surgery, especially PCs, LPCs, SMs, acylcarnitines, and BCAAs^30^. LPCs are generated from the turnover of PCs in the circulation by the pro-inflammatory phospholipase A_2_ (PLA_2_), and act as immunomodulating lipid mediators and reparation of cell membranes^31^. In the present study, multiple PCs, LPCs, their ratios, and SMs decreased after surgery. Overall, most of these significant ratios corresponded with unsaturated PC/saturated PC species. This may reflect a preference for saturated fatty acids over polyunsaturated fatty acids in the signaling of inflammatory processes derived from surgery^32^. PCs reduction is linked to the increased damaging activity of certain phospholipases^33^, although this did not mirror an increase of other molecular species of LPC by such enzymes. Similar findings were observed following bariatric surgery^34,35^, coronary artery bypass surgery^36^, and sepsis^37^. However, Fiamoncini *et al*.^38^ found the opposite pattern in SMs, with increased levels of saturated and monounsaturated species after gastric bypass surgery. In the current study, we found a great alteration in amino acid balance after surgery. A few of them had previously been reported in the literature. For instance, BCAAs, which increased after CRC surgery, were also raised after adrenalectomy in another work that presented a similar pattern^39^. There has been a decrease in BCAAs reported after bariatric surgery^38,40^, which may be related to an improvement in insulin activity^41^.

We found a prominent role in decreased LPCs and ratios associated with the severity of postoperative complications, suggesting a reduced turnover of LPC-PC. This hypothesis points out toward recycling PCs via the Lands cycle^31^. Interestingly, LPC16:0 and LPC18:0 displayed a remarkably inverse association with both CDC and CCI. We propose that postoperative plasma samples with a LPC18:2/PCa36:2 ratio below the cut-off of 0.084 µM/µM are a risk factor with a sevenfold risk for presenting major postoperative complications following CRC surgery and a fivefold risk of severe AL-related complications. A decreased concentration of LPC helped with the prediction of 28-day mortality in ICU patients with severe sepsis or septic shock^42^. Our results also agree with those of Matsuda *et al*.^22^, who reported a lower ratio of total LPC and PC (post/preoperative levels) in perioperative plasma of 43 patients undergoing CRC surgery with postoperative complications, defined as CDC ≥1. Furthermore, misbalances in LPC have been reported in several inflammatory states^43^, associated with oxidative stress^44^ and also have been suggested as biomarkers of colorectal cancer^45^.

The kynurenine/tryptophan ratio reached the strongest positive relationship with both CDC and CCI. This ratio reflects the catalysis of tryptophan to kynurenine by the enzymatic activity of tryptophan-2,3-dioxygenase/indoleamine 2-3-dioxygenase (TDO/IDO). IDO is associated with inflammation^46^ and plays a pivotal role in immune tolerance^47^, that might underlie future outcomes. It induces suppression of T cell proliferation, contributing to the pathophysiology of immunodeficiency and affecting the growth of several pathogens. Other studies have demonstrated an association of the kynurenine/tryptophan ratio with the severity of sepsis and outcomes in trauma and surgery ICU patients. The authors showed that patients who developed sepsis had increased kynurenine/tryptophan ratios compared to healthy controls^48^, as well as nonsurvivors versus survivors^49,50^. This ratio correlated with different severity scores of septic patients such as APACHE-II, Simplified Acute Physiology Score (SAPS-II), and Logistic Organ Dysfunction System^51,52^. The ratio has been suggested as a risk factor for disease severity and fatality in patients with bacteraemia^47^. Likewise, we propose a kynurenine/tryptophan ratio cut-off of 0.067 µM/µM for prescribing a ninefold risk of presenting CCI-based major complications following CRC surgery.

CDC and CCI were associated with several amino acid-related compounds, including threonine, putrescine, serine, methionine sulfoxide, and glutamine. Amino acid pathways have been found to be dysregulated in bacterial infection and to play an essential role in adaptative and innate immunity. An amino acid metabolism disorder in sepsis has also been demonstrated^53^, and several amino acid-related compounds had been previously associated with postoperative outcomes. Bednarz-Misa *et al*.^23^ studied serum metabolites involved in the arginine/nitric oxide pathway in 60 patients undergoing CRC surgery and found several changes in the early postoperative period. The group with major complications experienced a drop in asymmetric dimethylarginine concentration between 8 and 24–72 h after incision. Moreover, differences in ratios of other metabolites from the arginine/nitric oxide pathway such as arginine and symmetric dimethylarginine, were associated with specific postoperative complications, including AL, surgical site infection, and postoperative ileus. In the present study, such metabolites were statistically related to the CRC surgery, but they were not associated with the severity of postoperative complications.

Although our results indicate that using Δ-data may be a suitable approach for anticipating severe postoperative complications, the analysis of a metabolic signature in one postoperative sample should facilitate the application of our findings into clinical practice. In our study, we found that determining the concentration of LPC16:1/LPC16:0, kynurenine/tryptophan, hexadecanoylcarnitine, and LPC18:2/PCa36:2 in one postoperative plasma sample is the most suitable approach to be validated in larger independent cohorts. Even though some serious postoperative complications could occur before, deregulation in the concentration of these markers at day 4 could serve as an early warning to prompt reassessment and appropriate management of the patient. This would facilitate early decision-making to reduce failure to rescue, which we believe is more important than the ability to predict any complication. Failure to rescue is a quality metric defined as mortality following a postoperative complication as a consequence of delay or failure in recognizing and responding to that complication^54^. It has been proposed as an underlying factor in hospital variation in surgical mortality and occurs predominantly among patients who have more than one complication with a dose-response relationship as complications accrue. On the other hand, CCI has been proposed to be a major improvement in assessing morbidity in patients with multiple events. Therefore, the association of circulating markers with the CCI in the early postoperative period has the potential to identify patients at risk and facilitate early decision-making to reduce this fatal outcome^55,56^.

## Conclusion

Our study provides evidence of surgery-induced metabolic alterations associated with the severity of postoperative complications following CRC surgery, including AL. We provide quantitative insight on metabolic markers to measure vulnerability to postoperative morbidity that, after validation in larger cohorts, could be translated into risk factors in the clinical practice.

## Ethical approval

Yes. Ethical Committee of Hospital del Mar, Barcelona, Spain on 2 June 2015. 2015/6198/I.

## Sources of funding

This study was supported by research grant from Instituto de Salud Carlos III (FIS grant PI19/00643) and co-funded by the European Union. FMG was supported by Grant FJC2018-035791-I funded by MCIN/AEI/10.13039/501100011033. SM and MC were supported by Grant PID2020-115051RB-I00 funded by MCIN/AEI/10.13039/501100011033. SM and MC were supported by Grant 2017SGR1033 funded by AGAUR. MC was supported by ‘ICREA Academia’ award funded by ICREA Foundation.

## Author contribution

B.M.: term, conceptualization, methodology, resources, data curation, writing – original draft, writing – review and editing, and visualization; F.M.-G.: conceptualization, methodology, formal analysis, data curation, writing – original draft, writing – review and editing, and visualization; Ó.J.P.: conceptualization, methodology, formal analysis, data curation, writing – original draft, writing – review and editing, visualization, and supervision; S.M.: formal analysis, data curation, and writing – review and editing; S.M.: investigation, resources, data curation, writing – review and editing; X.M.: resources, data curation, writing – review and editing; M.P.: data curation, writing – review and editing; S.A.: data curation, writing – review and editing; S.S.: data curation, writing – review and editing; M.J.-T.: data curation, writing – review and editing; M.C.: investigation, resources, data curation, writing – review and editing; M.P.: term, conceptualization, methodology, resources, data curation, writing – original draft, writing – review and editing, visualization, supervision, project administration, and funding acquisition. All authors have approved the final draft and are responsible for the content.

## Conflicts of interest disclosure

The authors declare that they have no financial conflicts of interest with regard to the content of this report.

## Research registration unique identifying number (UIN)

1. Registry used: ClinicalTrials.gov.
2. Unique identifying number or registration ID: NCT02789709.
3. Hyperlink to specific registration: https://clinicaltrials.gov/ct2/results?cond=&term=NCT02789709&cntry=&state=&city=&dist=.

## Guarantor

All authors.

## Data availability statement

All original data are available upon reasonable request to the corresponding authors. The data are not publicly available since this could compromise the privacy of research participants.

## Supplementary Material

SUPPLEMENTARY MATERIAL
