# Supplementary material for: Circulating metabolic markers after surgery identify patients at risk for severe postoperative complications: a prospective cohort study in colorectal cancer
Source: Int J Surg. 2023 Dec 18;110(3):1493–501. doi: 10.1097/JS9.0000000000000965 (PMC10942180; doi:10.1097/JS9.0000000000000965)

**Online-Only Supplements**

**Supplementary Table 1.** Metabolite ratios and targeted metabolic processes (p. 2)

**Supplementary Table 2.** Differential metabolic markers between pre and postsurgery (pp. 3-5)

**Supplementary Fig. 1.** Partial least squares regression plots of actual and predicted (top) Clavien-Dindo classification and (bottom) Comprehensive Complication Index using the preoperative dataset (p. 6)

**Supplementary Fig. 2.** Partial least squares regression plots of actual and predicted (top) Clavien-Dindo classification and (bottom) Comprehensive Complication Index using the Δ-dataset (p. 7)

**Supplementary Fig. 3.** Biplots obtained from repeated double cross-validation-partial least squares analysis, indicating the relationship of the variables with (top) Clavien-Dindo classification and (bottom) Comprehensive Complication Index. Points corresponding with patients are in grayscale: the darker the color, the higher the postoperative index (p. 8)

**Supplementary Fig. 4.** Predictive ability of dichotomized metabolic markers for (A) Clavien-Dindo classification, (B) Comprehensive Complication Index, and (C) anastomotic leakage-related complications. The area under the curves (AUCs) from the receiver operating characteristic (ROC) analysis were extracted from adjusted logistic models (see section 2.3. Statistical analysis and Table 3) (p. 9)

**Supplementary Table 1.** Metabolite ratios and targeted metabolic processes.

| Metabolite Ratio | Metabolic Process |
| --- | --- |
| Kynurenine/tryptophan | Enzymatic activity of TDO/IDO |
| LPC16:1/PCa32:2 | Turnover between LPCs diacyl-PCs |
| LPC16:0/PCa32:0 | Turnover between LPCs and diacyl-PCs |
| LPC16:1/PCe32:2 | Turnover between LPCs and acyl-alkyl-PCs |
| LPC18:0/PCa36:0 | Turnover between LPCs and diacyl-PCs |
| LPC18:0/PCe36:0 | Turnover between LPCs and acyl-alkyl-PCs |
| LPC18:1/PCa36:1 | Turnover between LPCs and diacyl-PCs |
| LPC18:2/PCa36:2 | Turnover between LPCs and diacyl-PCs |
| LPC18:2/PCe36:2 | Turnover between LPCs and acyl-alkyl-PCs |
| LPC18:1/PCe36:1 | Turnover between LPCs and acyl-alkyl-PCs |
| LPC16:1/LPC16:0 | Balance between monounsaturated and saturated short chain LPCs |
| LPC18:2/LPC18:0 | Balance between polyunsaturated and saturated short chain LPCs |
| LPC26:1/LPC26:0 | Balance between monounsaturated and saturated long chain LPCs |
| LPC28:1/LPC28:0 | Balance between monounsaturated and long chain LPCs |
| PCa32:3/PCa32:0 | Balance between polyunsaturated and saturated diacyl-PCs |
| PCa36:4/PCa36:0 | Balance between polyunsaturated and saturated diacyl-PCs |
| PCa38:4/PCa38:0 | Balance between polyunsaturated and saturated diacyl-PCs |
| PCa42:4/PCa42:0 | Balance between polyunsaturated and saturated diacyl-PCs |
| PCe34:3/PCe34:0 | Balance between polyunsaturated and saturated acyl-alkyl-PCs |
| PCe36:4/PCe36:0 | Balance between polyunsaturated and saturated acyl-alkyl-PCs |
| PCe38:4/PCe38:0 | Balance between polyunsaturated and saturated acyl-alkyl-PCs |

Abbreviations: LPC, lysophosphatidylcholine; PC, phosphatidylcholine; TDO/IDO, tryptophan-2,3-dioxygenase/indoleamine 2-3-dioxygenase.

**Supplementary Table 2.** Differential metabolic markers between pre and postsurgery.

| Metabolic Marker | Presurgery (μM) | Postsurgery (μM) | *P^a^* | FDR | LR^b^ |
| --- | --- | --- | --- | --- | --- |
| Leucine | 113.35 ± 29.99 | 164.87 ± 48.48 | <0.001 | <0.001 | 1 |
| PCa34:4 | 0.62 ± 0.29 | 0.36 ± 0.17 | <0.001 | <0.001 | 2 |
| PCa34:2 | 188.55 ± 46.14 | 226.18 ± 46.67 | <0.001 | <0.001 | 3 |
| Methionine | 17.88 ± 5.30 | 25.49 ± 8.15 | <0.001 | <0.001 | 4 |
| Valerylcarnitine | 0.15 ± 0.11 | 0.62 ± 0.65 | <0.001 | <0.001 | 5 |
| PCa40:4 | 2.60 ± 0.85 | 1.9 ± 0.67 | <0.001 | <0.001 | 6 |
| PCa36:6 | 0.39 ± 0.19 | 0.23 ± 0.1 | <0.001 | <0.001 | 7 |
| PCe40:4 | 1.53 ± 0.38 | 1.17 ± 0.37 | <0.001 | <0.001 | 8 |
| PCa42:4 | 0.15 ± 0.05 | 0.11 ± 0.03 | <0.001 | <0.001 | 9 |
| PCe36:4 | 9.68 ± 2.86 | 7.08 ± 2.37 | <0.001 | <0.001 | 10 |
| PCe36:5 | 6.46 ± 2.10 | 4.68 ± 1.65 | <0.001 | <0.001 | 11 |
| PCe38:6 | 4.8 ± 1.46 | 3.55 ± 1.07 | <0.001 | <0.001 | 12 |
| PCe38:0 | 1.23 ± 0.47 | 0.89 ± 0.31 | <0.001 | <0.001 | 13 |
| PCa32:3/PCa32:0 | 0.03 ± 0.01 | 0.02 ± 0.01 | <0.001 | <0.001 | 14 |
| PCa42:5 | 0.25 ± 0.07 | 0.19 ± 0.05 | <0.001 | <0.001 | 15 |
| Butyrylcarnitine | 0.2 ± 0.13 | 0.32 ± 0.21 | <0.001 | <0.001 | 16 |
| Isoleucine | 67.63 ± 16.97 | 92.94 ± 26.76 | <0.001 | <0.001 | 17 |
| PCe36:4/PCe36:0 | 16.31 ± 5.54 | 11.59 ± 4.29 | <0.001 | <0.001 | 18 |
| PCa32:0 | 7.09 ± 1.74 | 8.3 ± 1.81 | <0.001 | <0.001 | 19 |
| LPC18:2/LPC18:0 | 1.04 ± 0.34 | 1.35 ± 0.43 | <0.001 | <0.001 | 20 |
| PCe38:4 | 8.35 ± 2.05 | 6.53 ± 2.02 | <0.001 | <0.001 | 21 |
| PCe34:3 | 2.84 ± 1.03 | 2.07 ± 0.92 | <0.001 | <0.001 | 22 |
| PCa42:2 | 0.24 ± 0.08 | 0.19 ± 0.06 | <0.001 | <0.001 | 23 |
| PCe40:6 | 3.26 ± 1.01 | 2.53 ± 0.81 | <0.001 | <0.001 | 24 |
| PCe40:5 | 2.59 ± 0.70 | 2.04 ± 0.60 | <0.001 | <0.001 | 25 |
| PCe40:3 | 0.73 ± 0.22 | 0.58 ± 0.17 | <0.001 | <0.001 | 26 |
| PCe42:2 | 0.45 ± 0.15 | 0.35 ± 0.12 | <0.001 | <0.001 | 27 |
| PCe38:3 | 2.53 ± 0.71 | 1.99 ± 0.57 | <0.001 | <0.001 | 28 |
| SM24:0 | 16.43 ± 5.61 | 12.67 ± 4.02 | <0.001 | <0.001 | 29 |
| PCe38:5 | 12.66 ± 3.25 | 10.07 ± 2.92 | <0.001 | <0.001 | 30 |
| PCe34:3/PCe34:0 | 4.27 ± 1.37 | 3.33 ± 1.06 | <0.001 | <0.001 | 31 |
| PCa42:6 | 0.38 ± 0.12 | 0.3 ± 0.11 | <0.001 | <0.001 | 32 |
| Valine | 184.46 ± 45.41 | 224.53 ± 58.17 | <0.001 | <0.001 | 33 |
| PCe42:4 | 0.56 ± 0.17 | 0.45 ± 0.17 | <0.001 | <0.001 | 34 |
| PCe36:3 | 3.67 ± 1.10 | 2.87 ± 0.97 | <0.001 | <0.001 | 35 |
| PCa34:1 | 131.88 ± 35.82 | 152.61 ± 38.50 | <0.001 | <0.001 | 36 |
| PCa40:5 | 5.75 ± 1.89 | 4.58 ± 1.53 | <0.001 | <0.001 | 37 |
| SM18:0 | 17.37 ± 6.44 | 19.98 ± 6.35 | <0.001 | 0.001 | 38 |
| PCa40:3 | 0.41 ± 0.15 | 0.33 ± 0.10 | <0.001 | <0.001 | 39 |
| SM20:2 | 0.37 ± 0.14 | 0.44 ± 0.14 | <0.001 | <0.001 | 40 |
| PCa40:2 | 0.27 ± 0.1 | 0.21 ± 0.07 | <0.001 | <0.001 | 41 |
| SM18:1 | 8.08 ± 3.15 | 8.92 ± 2.99 | 0.010 | 0.015 | 42 |
| LPC17:0 | 0.93 ± 0.47 | 0.65 ± 0.32 | <0.001 | <0.001 | 43 |
| SM(OH)22:2 | 8.26 ± 2.75 | 6.87 ± 2.10 | <0.001 | <0.001 | 44 |
| PCa42:4/PCa42:0 | 0.36 ± 0.10 | 0.32 ± 0.10 | <0.001 | <0.001 | 45 |
| PCa38:0 | 2.70 ± 0.99 | 2.22 ± 0.77 | <0.001 | <0.001 | 46 |
| Arginine | 53.71 ± 20.64 | 69.85 ± 27.93 | <0.001 | <0.001 | 47 |
| SM16:1 | 11.33 ± 3.74 | 11.66 ± 3.13 | 0.350 | 0.408 | 48 |
| SM(OH)22:1 | 10.13 ± 3.30 | 8.14 ± 2.64 | <0.001 | <0.001 | 49 |
| PCe42:3 | 0.65 ± 0.23 | 0.53 ± 0.22 | <0.001 | <0.001 | 50 |
| PCe40:2 | 1.08 ± 0.32 | 0.89 ± 0.27 | <0.001 | <0.001 | 51 |
| PCe38:2 | 1.2 ± 0.38 | 0.97 ± 0.29 | <0.001 | <0.001 | 52 |
| PCe42:5 | 1.62 ± 0.46 | 1.4 ± 0.42 | <0.001 | <0.001 | 53 |
| Phenylalanine | 52.95 ± 10.28 | 63.27 ± 17.89 | <0.001 | <0.001 | 54 |
| PCa38:3 | 32.52 ± 10.97 | 26.09 ± 8.74 | <0.001 | <0.001 | 55 |
| PCe44:4 | 0.3 ± 0.08 | 0.25 ± 0.08 | <0.001 | <0.001 | 56 |
| SM(OH)24:1 | 1.08 ± 0.37 | 0.87 ± 0.27 | <0.001 | <0.001 | 57 |
| PCa42:1 | 0.27 ± 0.09 | 0.22 ± 0.07 | <0.001 | <0.001 | 58 |
| PCe44:5 | 1.42 ± 0.52 | 1.21 ± 0.48 | <0.001 | 0.001 | 59 |
| PCa36:0 | 1.39 ± 0.58 | 1.04 ± 0.47 | <0.001 | <0.001 | 60 |
| PCa38:1 | 1.04 ± 0.38 | 0.83 ± 0.30 | <0.001 | <0.001 | 61 |
| PCe44:6 | 0.88 ± 0.27 | 0.74 ± 0.27 | <0.001 | <0.001 | 62 |
| Asparagine | 36.13 ± 9.29 | 42.51 ± 11.29 | <0.001 | <0.001 | 63 |
| PCa38:4 | 77.15 ± 22.07 | 65.21 ± 21.03 | <0.001 | <0.001 | 64 |
| PCe40:1 | 1.15 ± 0.47 | 0.95 ± 0.47 | <0.001 | <0.001 | 65 |
| Methionine sulfoxide | 0.71 ± 0.27 | 0.97 ± 0.51 | <0.001 | <0.001 | 66 |
| PCa38:5 | 30.35 ± 8.76 | 25.37 ± 7.46 | <0.001 | <0.001 | 67 |
| PCe34:2 | 4.45 ± 1.34 | 3.65 ± 1.23 | <0.001 | <0.001 | 68 |
| LPC16:0/PCa32:0 | 8.03 ± 4.57 | 5.59 ± 2.89 | <0.001 | <0.001 | - |
| SM26:0 | 0.17 ± 0.14 | 0.13 ± 0.04 | <0.001 | <0.001 | - |
| LPC18:0 | 14.47 ± 7.37 | 10.29 ± 5.38 | <0.001 | <0.001 | - |
| PCa32:2 | 0.92 ± 0.55 | 0.61 ± 0.36 | <0.001 | <0.001 | - |
| LPC16:1 | 1.54 ± 0.79 | 1.16 ± 0.57 | <0.001 | <0.001 | - |
| Putrescine | 0.09 ± 0.05 | 0.12 ± 0.07 | <0.001 | <0.001 | - |
| Glutamate | 68.53 ± 34.27 | 51.68 ± 26.38 | <0.001 | <0.001 | - |
| Threonine | 102.07 ± 30.74 | 121.08 ± 40.23 | <0.001 | <0.001 | - |
| LPC18:0/PCe36:0 | 25.8 ± 16.30 | 17.74 ± 11.97 | <0.001 | <0.001 | - |
| Kynurenine/Trp | 0.05 ± 0.02 | 0.07 ± 0.05 | <0.001 | <0.001 | - |
| Citrulline | 29.16 ± 11.78 | 23.8 ± 9.83 | <0.001 | <0.001 | - |
| Total DMA | 1.03 ± 0.33 | 1.22 ± 0.45 | <0.001 | <0.001 | - |
| PCa36:4/PCa36:0 | 98.52 ± 46.81 | 142 ± 120.70 | <0.001 | <0.001 | - |
| PCe32:1 | 1.29 ± 0.39 | 1.12 ± 0.33 | <0.001 | <0.001 | - |
| PCa40:6 | 22.54 ± 8.54 | 19.08 ± 6.54 | <0.001 | <0.001 | - |
| PCa42:0 | 0.44 ± 0.15 | 0.38 ± 0.13 | <0.001 | 0.001 | - |
| PCe42:1 | 0.42 ± 0.22 | 0.34 ± 0.18 | <0.001 | 0.001 | - |
| Lysine | 177.25 ± 36.96 | 192.85 ± 43.79 | 0.001 | 0.001 | - |
| LPC20:3 | 1.49 ± 0.60 | 1.24 ± 0.64 | 0.001 | 0.001 | - |
| Kynurenine | 1.96 ± 0.66 | 2.33 ± 1.14 | 0.001 | 0.001 | - |
| PCa38:6 | 66.27 ± 23.84 | 57.72 ± 20.89 | 0.001 | 0.002 | - |
| Taurine | 57.63 ± 27.73 | 46.71 ± 24.98 | 0.001 | 0.002 | - |
| LPC16:0 | 55.29 ± 31.27 | 44.97 ± 22.19 | 0.001 | 0.002 | - |
| LPC18:1 | 13.05 ± 5.70 | 11.04 ± 5.37 | 0.001 | 0.003 | - |
| PCa32:3 | 0.21 ± 0.08 | 0.19 ± 0.07 | 0.002 | 0.003 | - |
| Alanine | 286.15 ± 86.97 | 255.89 ± 81.15 | 0.002 | 0.003 | - |
| LPC20:4 | 5.43 ± 2.71 | 4.51 ± 2.40 | 0.002 | 0.003 | - |
| alpha-Aminoadipic acid | 1.01 ± 0.44 | 1.2 ± 0.61 | 0.002 | 0.003 | - |
| Octadecadienylcarnitine | 0.06 ± 0.04 | 0.05 ± 0.02 | 0.002 | 0.004 | - |
| SM26:1 | 0.41 ± 0.20 | 0.35 ± 0.12 | 0.002 | 0.004 | - |
| Serine | 92.26 ± 23.91 | 101.86 ± 29.99 | 0.002 | 0.004 | - |
| LPC16:1/PCe32:2 | 4.71 ± 3.08 | 3.76 ± 2.25 | 0.003 | 0.004 | - |
| Tyrosine | 55.16 ± 16.62 | 60.7 ± 15.98 | 0.003 | 0.005 | - |
| PCa36:1 | 29.09 ± 9.72 | 26.2 ± 7.95 | 0.004 | 0.006 | - |
| PCa28:1 | 1.89 ± 0.66 | 1.7 ± 0.58 | 0.004 | 0.006 | - |
| PCe34:0 | 0.69 ± 0.24 | 0.63 ± 0.16 | 0.005 | 0.008 | - |
| Creatinine | 76.73 ± 23.46 | 85.7 ± 35.14 | 0.005 | 0.008 | - |
| Acetylornithine | 1.1 ± 1.13 | 0.78 ± 0.67 | 0.005 | 0.009 | - |
| PCe38:1 | 0.47 ± 0.25 | 0.4 ± 0.20 | 0.009 | 0.014 | - |
| LPC18:1/PCe36:1 | 3.43 ± 1.63 | 2.98 ± 1.38 | 0.009 | 0.015 | - |
| PCe32:2 | 0.38 ± 0.16 | 0.34 ± 0.13 | 0.011 | 0.017 | - |
| SDMA | 0.66 ± 0.26 | 0.76 ± 0.41 | 0.012 | 0.018 | - |
| Tetradecenoylcarnitine | 0.09 ± 0.04 | 0.1 ± 0.04 | 0.016 | 0.024 | - |
| PCe44:3 | 0.12 ± 0.07 | 0.11 ± 0.06 | 0.016 | 0.025 | - |
| PCa36:5 | 10.35 ± 5.65 | 8.99 ± 4.53 | 0.016 | 0.025 | - |
| LPC24:0 | 0.57 ± 0.55 | 0.44 ± 0.38 | 0.017 | 0.026 | - |
| Histidine | 64.32 ± 12.9 | 60.31 ± 16.32 | 0.019 | 0.028 | - |
| PCe36:1 | 4 ± 1.12 | 3.76 ± 0.88 | 0.023 | 0.034 | - |
| LPC28:0 | 0.86 ± 0.99 | 0.66 ± 0.62 | 0.025 | 0.037 | - |
| PCe30:0 | 0.29 ± 0.17 | 0.25 ± 0.14 | 0.034 | 0.049 | - |

*^a^*Models adjusted for age, sex, BMI smoking, alcoholism, arterial hypertension, diabetes mellitus, dyslipidemia, anticoagulant therapy, duration of surgery, surgical approach (laparoscopic versus open) and type of operation (colectomy versus proctectomy). ^b^Loading rank of dCV multilevel-Partial Least Square. Only significant auto-selected variables are numbered. Abbreviations: LPC, lysophosphatidylcholine; PC, phosphatidylcholine; SDMA, symmetric dimethylarginine; SM, sphingomyeline; Total DMA, total dimethylarginine.

**Supplementary Fig. 1.** Partial least squares regression plots of actual and predicted (top) Clavien-Dindo classification and (bottom) Comprehensive Complication Index using the preoperative dataset.


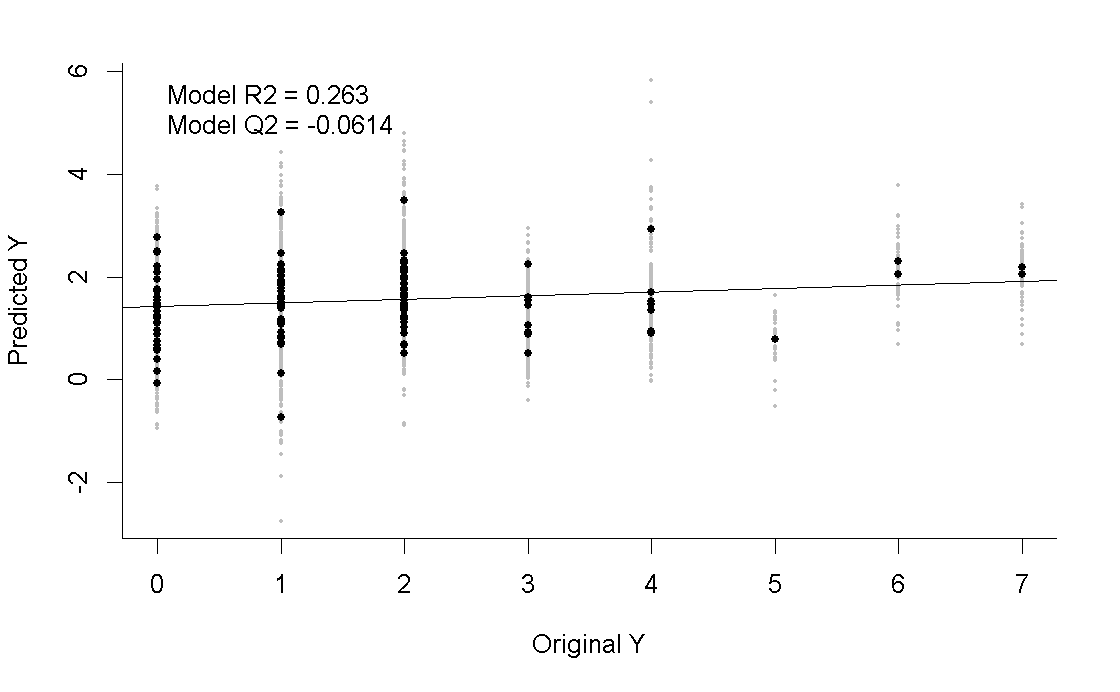

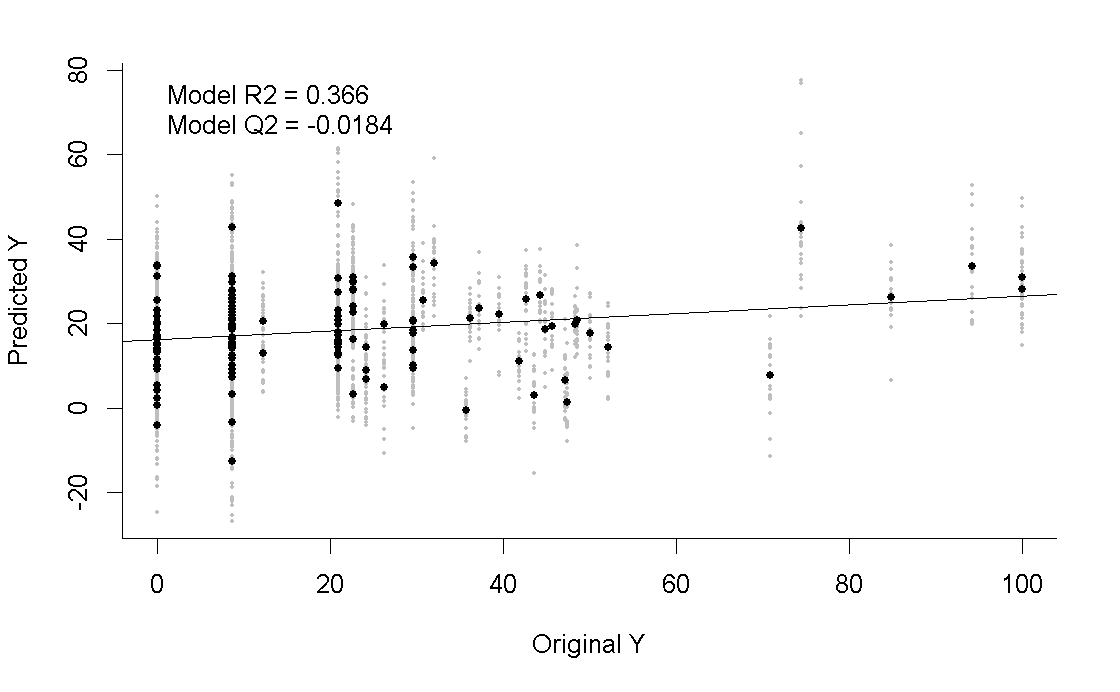


**Supplementary Fig. 2.** Partial least squares regression plots of actual and predicted (top) Clavien-Dindo classification and (bottom) Comprehensive Complication Index using the Δ-dataset.


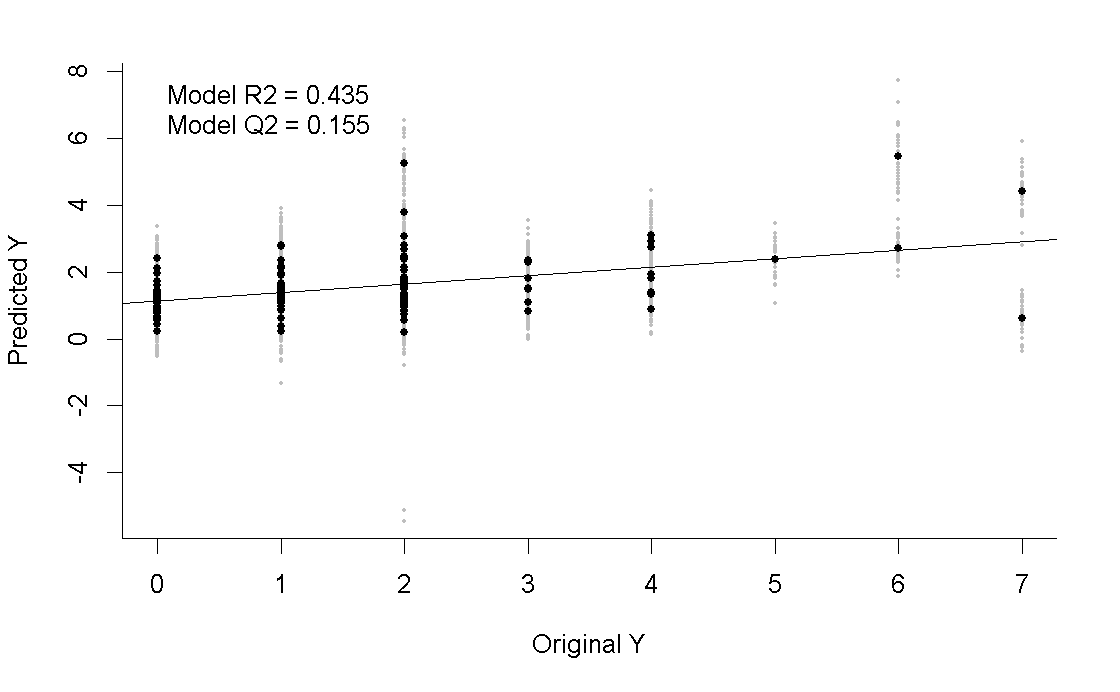


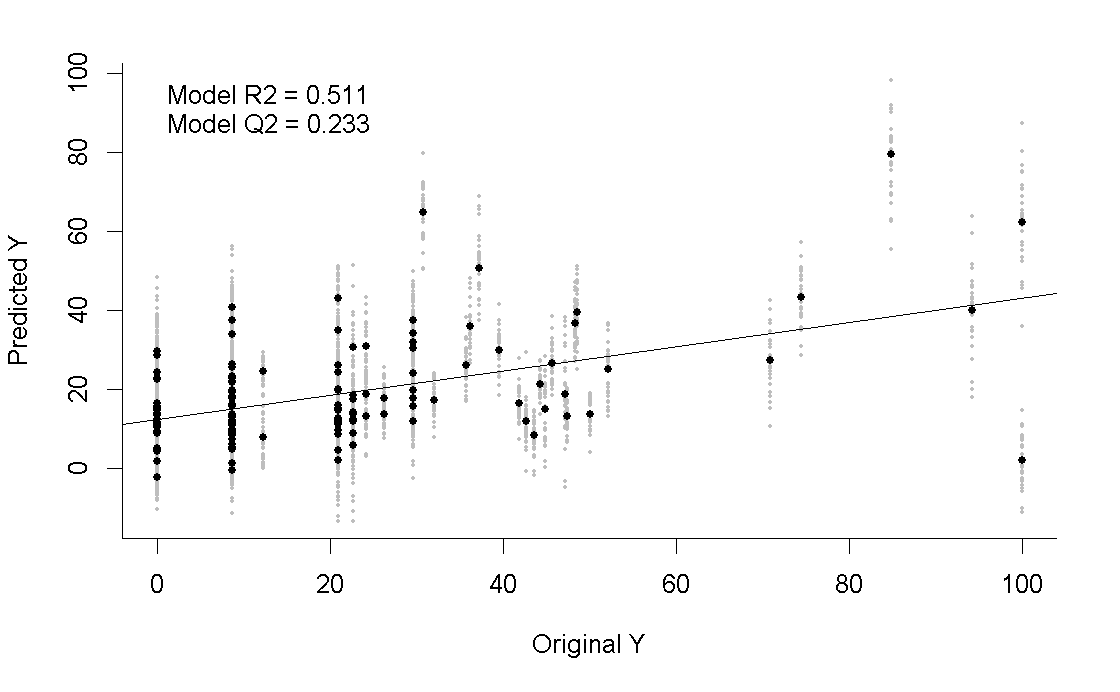


**Supplementary Fig. 3.** Biplots obtained from repeated double cross-validation-partial least squares analysis, indicating the relationship of the variables with (top) Clavien-Dindo classification and (bottom) Comprehensive Complication Index. Points corresponding with patients are in grayscale: the darker the color, the higher the postoperative index.


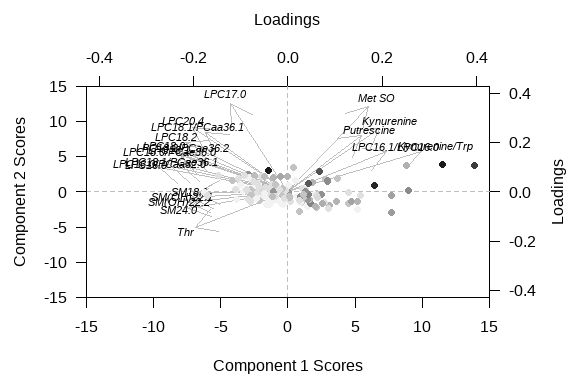

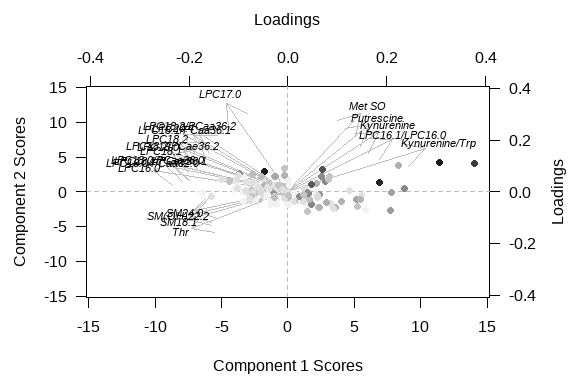


**Supplementary Fig. 4.** Predictive ability of dichotomized metabolic markers for (A) Clavien-Dindo classification, (B) Comprehensive Complication Index, and (C) anastomotic leakage-related complications. The area under the curves (AUCs) from the receiver operating characteristic (ROC) analysis were extracted from adjusted logistic models (see section 2.3. Statistical analysis and Table 3).


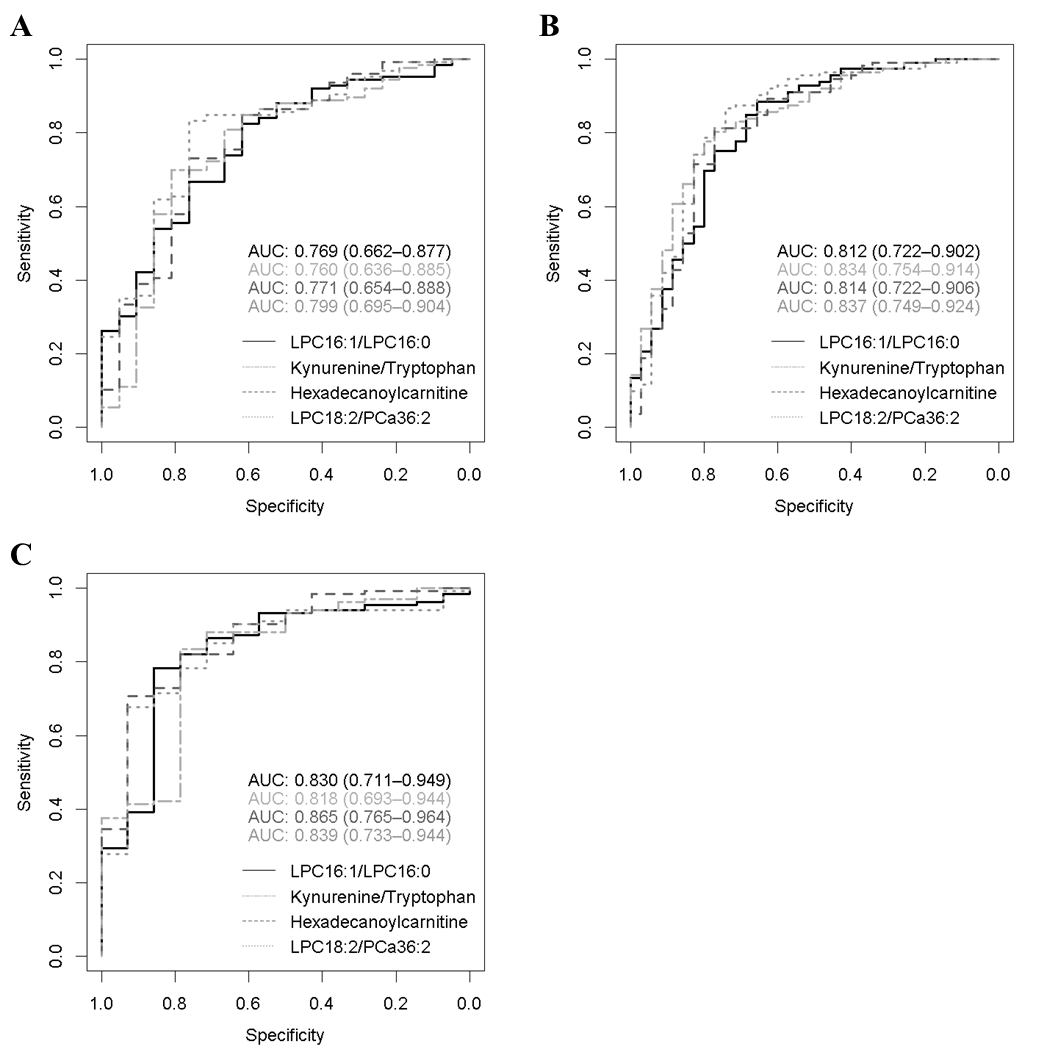

Supplement: SUPPLEMENTARY MATERIAL [file js9-110-1493-s002.docx]
